# Genome-Wide Study of Gene Variants Associated with Differential Cardiovascular Event Reduction by Pravastatin Therapy

**DOI:** 10.1371/journal.pone.0038240

**Published:** 2012-05-30

**Authors:** Dov Shiffman, Stella Trompet, Judy Z. Louie, Charles M. Rowland, Joseph J. Catanese, Olga A. Iakoubova, Todd G. Kirchgessner, Rudi G. J. Westendorp, Anton J. M. de Craen, P. Eline Slagboom, Brendan M. Buckley, David J. Stott, Naveed Sattar, James J. Devlin, Christopher J. Packard, Ian Ford, Frank M. Sacks, J. Wouter Jukema

**Affiliations:** 1 Celera Inc., Alameda, California, United States of America; 2 Department of Cardiology, Leiden University Medical Center, Leiden, The Netherlands; 3 Department of Gerontology and Geriatrics, Leiden University Medical Center, Leiden, The Netherlands; 4 Research and Development, Bristol-Myers Squibb, Princeton, New Jersey, United States of America; 5 Department of Molecular Epidemiology, Leiden University Medical Center, Leiden, The Netherlands; 6 Department of Pharmacology and Therapeutics, University College Cork, Cork, Ireland; 7 Division of Cardiovascular and Medical Sciences, University of Glasgow, Glasgow, United Kingdom; 8 Glasgow Cardiovascular Research Center, Faculty of Medicine, British Heart Foundation, University of Glasgow, Glasgow, United Kingdom; 9 Robertson Centre for Biostatistics, University of Glasgow, Glasgow, United Kingdom; 10 Brigham and Women’s Hospital, Harvard Medical School, Boston, Massachusetts, United States; 11 Durrer Center for Cardiogenetic Research, Amsterdam, The Netherlands; 12 Interuniversity Cardiology Institute of the Netherlands, Utrecht, The Netherlands; Universitätsklinikum Schleswig-Holstein - Campus Luebeck, Germany

## Abstract

Statin therapy reduces the risk of coronary heart disease (CHD), however, the person-to-person variability in response to statin therapy is not well understood. We have investigated the effect of genetic variation on the reduction of CHD events by pravastatin. First, we conducted a genome-wide association study of 682 CHD cases from the Cholesterol and Recurrent Events (CARE) trial and 383 CHD cases from the West of Scotland Coronary Prevention Study (WOSCOPS), two randomized, placebo-controlled studies of pravastatin. In a combined case-only analysis, 79 single nucleotide polymorphisms (SNPs) were associated with differential CHD event reduction by pravastatin according to genotype (P<0.0001), and these SNPs were analyzed in a second stage that included cases as well as non-cases from CARE and WOSCOPS and patients from the PROspective Study of Pravastatin in the Elderly at Risk/PHArmacogenomic study of Statins in the Elderly at risk for cardiovascular disease (PROSPER/PHASE), a randomized placebo controlled study of pravastatin in the elderly. We found that one of these SNPs (rs13279522) was associated with differential CHD event reduction by pravastatin therapy in all 3 studies: P = 0.002 in CARE, P = 0.01 in WOSCOPS, P = 0.002 in PROSPER/PHASE. In a combined analysis of CARE, WOSCOPS, and PROSPER/PHASE, the hazard ratio for CHD when comparing pravastatin with placebo decreased by a factor of 0.63 (95% CI: 0.52 to 0.75) for each extra copy of the minor allele (P = 4.8×10^−7^). This SNP is located in DnaJ homolog subfamily C member 5B (DNAJC5B) and merits investigation in additional randomized studies of pravastatin and other statins.

## Introduction

Statins, inhibitors of 3-hydroxy-3-methylglutaryl coenzyme A reductase (HMGCR), are widely prescribed to reduce low-density lipoprotein cholesterol (LDL-C) levels and cardiovascular events. In an analysis of 14 randomized clinical trials, statin therapy was associated with about 20% reduction of major cardiovascular events for each mmol/L (38.7 mg/dL) reduction of LDL-C [1]. Although statins are the most prescribed class of drugs and therapy is generally associated with LDL cholesterol lowering of 22–34%, individual variability in response to statin therapy has been noted. Recent research provides evidence that genetic variation contributes to this variable drug response [2], [3].

Multiple studies investigated whether genetic variants are associated with differential LDL-C reduction by statin therapy [4]. Evidence from several studies [5]–[7] suggests that the ε3 allele of *APOE* is associated with differential LDL-C lowering by statin therapy. Additionally, variants of the HMGCR gene have been also been shown to be associated with differential LDL-C reduction by statin treatment [6], [8], [9]. Several studies have reported an association between a *KIF6* variant (rs20455) and differential event reduction by pravastatin [10], [11] or intensive atorvastatin therapy [12], however, others found no association between rs20455 and differential event reduction from simvastatin [13] or rosuvastatin therapy [14].

To investigate the effect of genetic variation on the reduction of CHD events by pravastatin we conducted a genome wide association study (GWAS) in two large randomized controlled trials that used the same dose of pravastatin: Cholesterol and Recurrent Events (CARE) trial, and the West of Scotland Coronary Prevention Study (WOSCOPS) trial and replicated our findings in a third randomized control trial of pravastatin: PROspective Study of Pravastatin in the Elderly at Risk/PHArmacogenomic study of Statins in the Elderly at risk for cardiovascular disease (PROSPER/PHASE).

## Results

**Figure 1:**
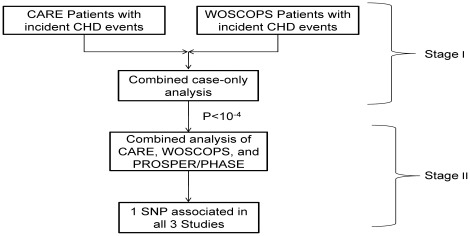
Study design. A flow chart outlining the patients and SNPs investigated in the 2 stages of the study.

A summary of the baseline characteristics of the patients included in the genetic analyses of CARE, WOSCOPS, and PROSPER is provided in Table 1. The first stage of this investigation included patients drawn from the CARE and WOSCOPS studies who had had an on-study CHD event (see strategy outline in Figure 1).

**Table 1:** Baseline characteristics of study participants.

	CARE			WOSCOPS			PROSPER/PHASE	
	Events(n = 711)	No Events(n = 2398)		Events(n = 522)	No Events(n = 4909)		Events(n = 590)	No Events(n = 4654)
Men, n (%)	618 (87)	2065 (86)		522 (100)	4909 (100)		356 (60)	2168 (47)
Age, years	58.3±9	58.±9		56.3±5	54.6±6		75.7±3	75.3±3
Body mass index, kg/m^2^	27.9±5	27.5±4		26.0±3	25.9±3		27.0±4	26.8±4
Current Smokers, n (%)	140 (20)	372 (16)		436 (84)	3815 (78)		152 (26)	1240 (27)
History of diabetes, n (%)	136 (19)	290 (12)		11 (2)	56 (1)		83 (14)	461 (10)
History of hypertension, n (%)	335 (47)	993 (41)		118 (23)	730 (15)		361 (61)	2896 (62)
LDL cholesterol, mg/dL	140.0±15	138.6±15		194.0±18	192.2±17		143.1±27	146.9±31
HDL cholesterol, mg/dL	38.2±8	38.8±9		41.5±9	44.3±9		46.4±12	50.3±16
Total cholesterol, mg/dL	209.6±17	208.±17		273.6±24	272.0±23		216.6±31	220.4±35
Triglycerides, mg/dL	158.2±60	156.4±6		174.9±74	161.1±68		141.7±62	132.9±62
Self-reported ethnicity				NA	NA		NA	NA
Caucasian, n (%)	667 (93.8)	2246 (93.7)						
African American, n (%)	18 (2.5)	60 (2.5)						
Hispanic, n (%)	20 (2.8)	65 (2.7)						
Asian, Pacific Islander, other, n (%)	6 (0.8)	27 (1.1)						

± values are standard deviation.

**Figure 2:**
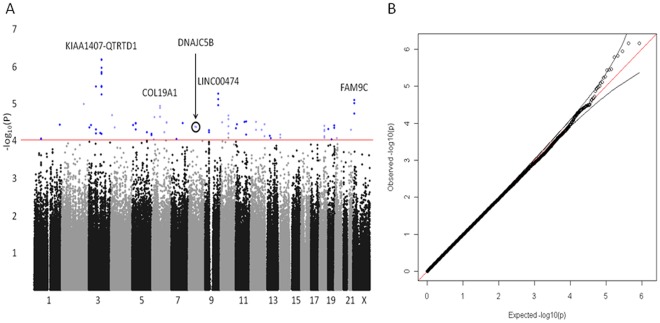
P-values from a combined case-only analysis of CARE and WOSCOPS. Panel A: A Manhattan plot of the P values from the combined case-only analysis of CARE and WOSCOPS. The x-axis corresponds to the chromosomal position for 22 chromosomes and the X chromosome. The Y axis corresponds to the negative base 10 logarithm of the P value. The red line indicates P = 10^−4^ and SNPs above this line are indicated in blue. Gene symbols are indicated above loci with P<10^-5^ in the combined analysis of CARE and WOSCOPS. The location of DNAJC5B is also indicated. Panel B: A quantile-quantile plot of the observed vs. expected P values. The black lines indicate the 95% CI associated with the expectation that, under the hypothesis of no association, the values will fall on the red diagonal.

Using a case-only analysis of CARE and WOSCOPS we determined the Synergy Index, an estimate of the interaction between pravastatin therapy and genotype for each SNP [15]. The P values for the combined Synergy Index from the CARE and WOSCOPS studies were calculated and plotted according to chromosomal position (Figure 2). Loci that included SNPs with low combined P values (<10^−5^) were found around *QTRRTD1* and *KIAA1407* on chromosome 3, near *LINC00474* on chromosome 9, and near *FAM9C* on chromosome X (Table 2). Overall we observed 79 SNPs that were nominally (P<10^−4^) associated with differential event reduction by pravastatin therapy (Table 2). These 79 SNPs clustered in 45 loci, where a locus is defined by associated SNPs that were within 100 kb of each other. The 45 loci were all >300 kb apart or on different chromosomes. None of these SNPs was in or near a gene that had been previously reported to be associated with CHD, involved in cholesterol metabolism, or involved in pravastatin metabolism. Furthermore, none of these SNPs was associated with baseline LDL-C levels among cases (P>0.05 in a combined analysis of CARE and WOSCOPS prior to adjustment for testing 45 loci), or with change in LDL-C levels in the pravastatin group of CARE (P>0.05, prior to adjustment for testing 45 loci).

**Table 2:** Most significant SNPs associated with differential response to pravastatin therapy in a case-only combined analysis of CARE and WOSCOPS.

SNP	CHR	Position	SI (95%CI)	P value	A1	A2	A1 Frq.	Gene	Next closest gene
rs7521242	1	61803889	0.71 (0.59 – 0.84)	8.97E-05	A	G	0.45	NFIA	TM2D1
rs9436636	1	61812773	1.43 (1.20 – 1.71)	9.09E-05	A	G	0.40	NFIA	TM2D1
rs2492367	1	231906589	0.54 (0.40 – 0.72)	3.77E-05	A	G	0.11	TSNAX-DISC1	DISC1
rs10189905	2	199679110	0.49 (0.36 – 0.67)	1.05E-05	C	A	0.10	.	SATB2
rs1450092	3	7506105	0.61 (0.48 – 0.77)	4.45E-05	G	A	0.18	GRM7	LOC100288428
rs1349282	3	22648091	0.68 (0.57 – 0.82)	3.89E-05	A	G	0.42	.	UBE2E2
rs1917527	3	65580498	0.69 (0.57 – 0.83)	6.53E-05	A	C	0.39	MAGI1	SLC25A26
rs1524962	3	65584681	0.61 (0.50 – 0.75)	3.57E-06	A	G	0.26	MAGI1	SLC25A26
rs7629574	3	65587758	0.66 (0.54 – 0.81)	5.05E-05	A	C	0.27	MAGI1	SLC25A26
rs7625204	3	102058887	1.54 (1.25 – 1.90)	6.32E-05	A	G	0.22	.	ZPLD1
rs16861467	3	113752500	0.45 (0.32 – 0.62)	1.65E-06	G	A	0.10	KIAA1407	QTRTD1
rs2129571	3	113753571	0.47 (0.34 – 0.65)	3.50E-06	A	G	0.10	KIAA1407	QTRTD1
rs9859901	3	113769097	0.46 (0.33 – 0.63)	1.50E-06	A	C	0.10	KIAA1407	QTRTD1
rs10155047	3	113772952	0.45 (0.33 – 0.62)	1.13E-06	A	G	0.10	KIAA1407	QTRTD1
rs16861476	3	113783883	0.43 (0.31 – 0.60)	6.86E-07	C	A	0.10	QTRTD1	KIAA1407
rs13314266	3	113797117	0.45 (0.32 – 0.64)	5.84E-06	G	A	0.08	QTRTD1	KIAA1407
rs3732788	3	113804859	0.45 (0.32 – 0.61)	6.93E-07	G	A	0.10	QTRTD1	KIAA1407
rs13318232	3	113811688	0.47 (0.35 – 0.65)	3.64E-06	G	A	0.10	.	QTRTD1
rs4682522	3	113829346	0.52 (0.37 – 0.72)	6.66E-05	C	A	0.09	.	DRD3
rs13137776	4	11464975	0.66 (0.54 – 0.80)	2.12E-05	G	A	0.33	.	HS3ST1
rs7671659	4	34353548	1.64 (1.29 – 2.07)	4.53E-05	A	G	0.16	.	ARAP2
rs9857	5	10680547	0.65 (0.53 – 0.80)	3.84E-05	G	A	0.24	DAP	ANKRD33B
rs4242084	5	34259360	0.22 (0.11 – 0.45)	3.41E-05	A	C	0.03	.	C1QTNF3-AMACR
rs4866354	5	34259427	0.22 (0.11 – 0.45)	3.50E-05	G	A	0.03	.	C1QTNF3-AMACR
rs4626316	5	34278426	0.23 (0.11 – 0.47)	5.03E-05	A	G	0.03	.	C1QTNF3-AMACR
rs12659030	5	134490348	1.51 (1.24 – 1.85)	6.01E-05	A	G	0.25	.	LOC340073
rs17076974	5	173619944	1.43 (1.20 – 1.71)	6.68E-05	A	G	0.48	.	HMP19
rs7705993	5	173620286	1.43 (1.20 – 1.71)	7.09E-05	G	A	0.48	.	HMP19
rs1544214	6	15990916	1.41 (1.19 – 1.67)	9.29E-05	A	G	0.40	.	MYLIP
rs7751843	6	22201594	1.91 (1.42 – 2.58)	2.38E-05	A	G	0.09	.	LINC00340
rs7742508	6	70777350	2.30 (1.58 – 3.35)	1.34E-05	A	G	0.05	COL19A1	COL9A1
rs3793048	6	70784746	2.05 (1.47 – 2.85)	2.33E-05	A	C	0.08	COL19A1	COL9A1
rs9446187	6	70786466	2.11 (1.51 – 2.96)	1.20E-05	A	G	0.08	COL19A1	COL9A1
rs2505039	6	110305904	0.69 (0.58 – 0.83)	6.02E-05	A	G	0.45	.	GPR6
rs9375683	6	130263591	1.55 (1.26 – 1.91)	3.36E-05	G	A	0.21	.	L3MBTL3
rs1538185	6	130267753	1.60 (1.28 – 1.99)	3.24E-05	A	G	0.20	.	L3MBTL3
rs2941528	7	47494959	0.67 (0.54 – 0.82)	9.18E-05	A	G	0.26	TNS3	C7orf65
rs1615197	7	105273458	1.45 (1.21 – 1.72)	3.41E-05	G	A	0.40	ATXN7L1	EFCAB10
rs12155847	8	58702529	1.48 (1.23 – 1.79)	4.28E-05	G	A	0.34	.	FAM110B
rs13279522	8	66974252	0.60 (0.48 – 0.77)	4.41E-05	G	A	0.17	DNAJC5B	TRIM55
rs7863577	9	36539122	0.51 (0.37 – 0.71)	5.37E-05	A	C	0.09	.	MELK
rs2265346	9	36635409	0.53 (0.39 – 0.72)	6.10E-05	C	A	0.10	MELK	MIR4475
rs10429616	9	118518733	1.50 (1.26 – 1.80)	7.87E-06	G	A	0.44	.	LINC00474
rs2418412	9	118541068	1.49 (1.25 – 1.79)	1.14E-05	G	A	0.43	.	LINC00474
rs2157673	9	118559109	1.55 (1.28 – 1.88)	5.58E-06	G	A	0.33	.	LINC00474
rs7910196	10	13810059	1.68 (1.31 – 2.14)	3.29E-05	G	A	0.15	FRMD4A	PRPF18
rs3998860	10	70405855	0.62 (0.50 – 0.78)	4.21E-05	A	G	0.19	TET1	CCAR1
rs7913568	10	70410630	0.61 (0.49 – 0.77)	2.71E-05	G	A	0.19	TET1	CCAR1
rs10762236	10	70431420	0.61 (0.48 – 0.77)	2.14E-05	C	A	0.19	TET1	CCAR1
rs10740308	10	70454896	0.63 (0.50 – 0.79)	6.58E-05	A	C	0.19	.	TET1
rs7901888	10	70455490	0.63 (0.50 – 0.79)	8.45E-05	A	C	0.19	.	TET1
rs231358	11	2702513	1.44 (1.21 – 1.72)	4.73E-05	G	A	0.51	KCNQ1	KCNQ1DN
rs17347854	11	11247842	0.60 (0.47 – 0.77)	3.73E-05	G	A	0.16	.	GALNTL4
rs17138705	11	79366322	0.57 (0.43 – 0.74)	3.19E-05	A	G	0.14	.	ODZ4
rs10831415	11	95466159	1.47 (1.23 – 1.77)	3.08E-05	G	A	0.41	.	FAM76B
rs1016030	11	95472693	1.45 (1.21 – 1.74)	7.02E-05	G	A	0.41	.	FAM76B
rs2335451	12	49664786	0.65 (0.54 – 0.80)	3.21E-05	A	C	0.28	TUBA1C	PRPH
rs10875941	12	49667921	0.66 (0.54 – 0.81)	5.17E-05	G	A	0.28	.	TUBA1C
rs10778050	12	100768827	1.44 (1.21 – 1.71)	5.19E-05	A	G	0.47	SLC17A8	SCYL2
rs2656824	12	127815086	0.67 (0.54 – 0.82)	7.80E-05	A	G	0.26	.	LOC440117
rs2593270	12	127815178	0.66 (0.54 – 0.80)	3.66E-05	A	G	0.26	.	LOC440117
rs238272	13	42933855	1.45 (1.21 – 1.74)	7.48E-05	A	G	0.31	.	AKAP11
rs2273816	13	50294056	1.61 (1.27 – 2.05)	8.71E-05	A	G	0.15	KPNA3	EBPL
rs3759607	14	23566143	1.99 (1.42 – 2.79)	7.03E-05	G	A	0.07	C14orf119	ACIN1
rs2069542	14	23900794	1.64 (1.28 – 2.11)	8.51E-05	A	G	0.15	MYH7	MIR208B
rs2196180	18	44861262	1.48 (1.22 – 1.81)	8.46E-05	A	G	0.26	.	IER3IP1
rs3861810	18	44864122	1.49 (1.22 – 1.81)	7.69E-05	G	A	0.26	.	IER3IP1
rs1594887	18	44892369	1.51 (1.24 – 1.84)	4.07E-05	G	A	0.26	.	IER3IP1
rs2060411	18	44918564	1.50 (1.23 – 1.83)	5.63E-05	A	G	0.26	.	IER3IP1
rs9807521	18	44935746	1.51 (1.24 – 1.84)	5.17E-05	A	C	0.26	.	IER3IP1
rs312929	19	3273188	0.68 (0.56 – 0.82)	4.95E-05	G	A	0.38	CELF5	NCLN
rs17716275	19	29577230	2.31 (1.52 – 3.53)	9.39E-05	A	G	0.05	.	LOC100505835
rs2657940	19	52863836	1.55 (1.25 – 1.91)	4.63E-05	A	G	0.22	ZNF610	ZNF880
rs2263901	19	52863967	1.55 (1.26 – 1.91)	3.96E-05	G	A	0.23	ZNF610	ZNF880
rs6075209	20	17415219	0.69 (0.58 – 0.83)	8.80E-05	A	G	0.41	PCSK2	BFSP1
rs2281122	22	35943984	1.61 (1.28 – 2.03)	5.22E-05	A	G	0.18	RASD2	MB
rs4830819	X	13044003	0.58 (0.46 – 0.74)	8.23E-06	G	A	0.47	.	FAM9C
rs2008165	X	13049032	0.59 (0.47 – 0.75)	1.90E-05	G	A	0.47	.	FAM9C
rs12556591	X	13050626	0.58 (0.46 – 0.74)	1.00E-05	G	A	0.47	.	FAM9C

**Figure 3:**
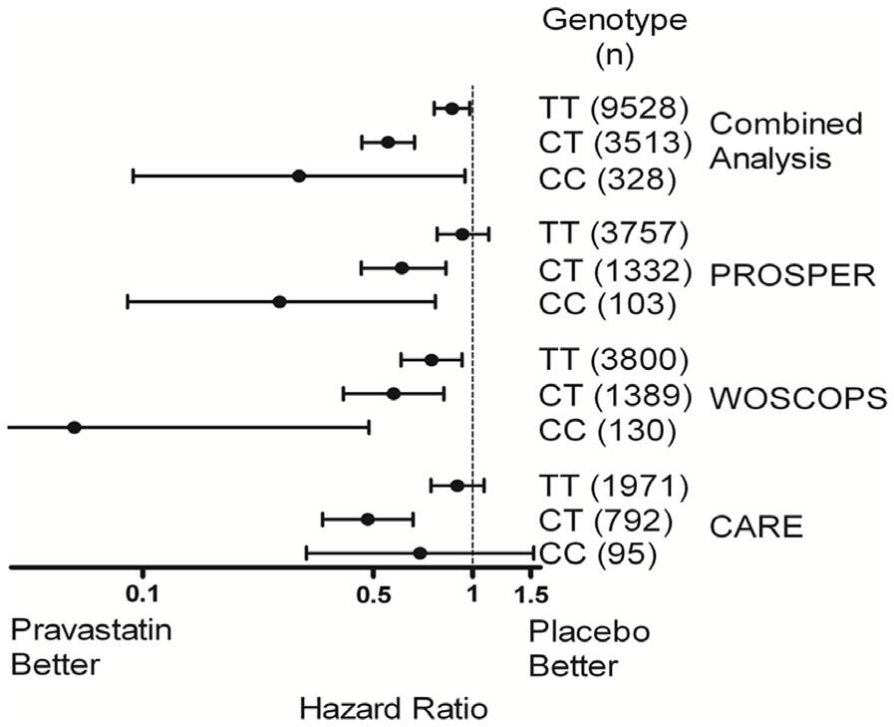
Risk reduction according to genotype for rs13279522. Risk estimate and 95% confidence intervals are indicated for each genotype group in each study, as well as for the combined analysis of the studies. The number of individuals in each group is indicated.

In the second stage of this investigation, we analyzed 74 of these 79 SNPs in PROSPER/PHASE (genotypes were not available in PROSPER/PHASE for the remaining 5 SNPs). We have also determined the genotypes for the remaining patients from CARE and WOSCOPS (with or without CHD events) for most of these 74 SNPs and conducted an analysis of these 74 SNPs in the combined CARE, WOSCOPS, and PROSPER/PHASE studies (Table 3). We found that one SNP (rs13279522) was associated with differential event reduction in all three studies (combined P for interaction between CHD events and pravastatin therapy = 4.8×10^−7^). The minor allele (C) of the rs13279522 SNP had a frequency of 16% among 2775 self-described Caucasian of the CARE study and the genotype distribution of this SNPs did not deviate from Hardy-Weinberg equilibrium expectations (P = 0.3). The frequency of the C allele was 61% among 76 self-described African Americans in CARE, 23% among 83 self-described Hispanics and 19% among 31 self-described Asians or Pacific islanders. We estimated the risk reduction by pravastatin in each genotype group in each study and in a combined analysis of all three studies (Figure 3). The risk reduction by pravastatin among minor homozygotes was 70% (95%CI 5% to 91%, P = 0.04) among heterozygotes was 45% (95%CI 33% to 54%, P = 2.9×10^−10^) and among major homozygotes was 13% (95%CI 2% to 24%, P = 0.02). This SNP was not associated with baseline LDL-C levels among cases and non-cases in CARE (P>0.7) or in PROSPER/PHASE (P>0.18). Neither was it associated with change in LDL-C from baseline to 3 month visit in the pravastatin groups of CARE (P>0.4) or PROSPER/PHASE (P>0.09).

**Table 3:** Individual and Combined analysis of 54 SNPs in CARE, WOSCOPS, and PROSPER/PHASE.

			Combined Analysis		CARE		WOSCOPS		PROSPER	
SNP	LD SNP	r^2^	SI (95%CI)	P value	SI (95%CI)	P value	SI (95%CI)	P value	SI (95%CI)	P value
rs13279522			0.63 (0.52 – 0.75)	4.84E-07	0.63 (0.47 – 0.84)	1.87E-03	0.63 (0.44 – 0.90)	1.12E-02	0.62 (0.46 – 0.84)	2.39E-03
rs3732788			0.61 (0.48 – 0.77)	3.95E-05	0.55 (0.37 – 0.82)	2.80E-03	0.54 (0.33 – 0.87)	1.08E-02	0.72 (0.49 – 1.07)	1.02E-01
rs13318232			0.62 (0.49 – 0.78)	5.66E-05	0.61 (0.42 – 0.89)	1.11E-02	0.49 (0.30 – 0.79)	3.19E-03	0.72 (0.49 – 1.07)	1.03E-01
rs1538185			1.41 (1.19 – 1.67)	6.58E-05	1.61 (1.22 – 2.12)	7.00E-04	1.26 (0.90 – 1.75)	1.77E-01	1.33 (1.01 – 1.75)	4.22E-02
rs16861476	rs3732788	CW, 1	0.62 (0.49 – 0.79)	9.61E-05	0.55 (0.37 – 0.82)	2.80E-03	0.54 (0.33 – 0.87)	1.08E-02	0.77 (0.52 – 1.15)	2.00E-01
rs9859901			0.63 (0.49 – 0.80)	1.17E-04	0.59 (0.40 – 0.87)	6.91E-03	0.50 (0.31 – 0.82)	5.37E-03	0.77 (0.52 – 1.14)	1.90E-01
rs2505039			0.77 (0.67 – 0.88)	1.21E-04	0.69 (0.56 – 0.86)	8.36E-04	0.78 (0.60 – 1.02)	6.83E-02	0.85 (0.68 – 1.06)	1.53E-01
rs10155047			0.62 (0.49 – 0.79)	1.26E-04	0.59 (0.40 – 0.86)	6.05E-03	0.50 (0.31 – 0.80)	4.45E-03	0.77 (0.52 – 1.14)	1.90E-01
rs13314266			0.61 (0.47 – 0.79)	1.45E-04	0.63 (0.41 – 0.94)	2.58E-02	0.46 (0.28 – 0.77)	3.08E-03	0.71 (0.46 – 1.10)	1.25E-01
rs9375683			1.36 (1.16 – 1.60)	1.57E-04	1.54 (1.18 – 2.00)	1.37E-03	1.25 (0.91 – 1.71)	1.76E-01	1.29 (0.99 – 1.68)	6.02E-02
rs16861467			0.60 (0.46 – 0.80)	4.21E-04	0.59 (0.40 – 0.87)	8.43E-03	0.45 (0.27 – 0.73)	1.46E-03	0.76 (0.51 – 1.12)	1.59E-01
rs4682522			0.65 (0.51 – 0.83)	4.47E-04	0.63 (0.42 – 0.93)	2.07E-02	0.56 (0.35 – 0.90)	1.72E-02	0.74 (0.50 – 1.10)	1.42E-01
rs6075209†			0.77 (0.65 – 0.90)	1.01E-03	0.66 (0.53 – 0.83)	3.42E-04	0.83 (0.61 – 1.12)	2.20E-01	0.84 (0.68 – 1.05)	1.30E-01
rs12659030			1.28 (1.10 – 1.48)	1.19E-03	1.26 (0.99 – 1.62)	6.54E-02	1.42 (1.07 – 1.87)	1.38E-02	1.19 (0.93 – 1.51)	1.63E-01
rs4626316	rs7701604	P, 1	0.45 (0.27 – 0.73)	1.20E-03	0.41 (0.22 – 0.74)	3.13E-03	0.47 (0.17 – 1.31)	1.50E-01	0.73 (0.16 – 3.35)	6.81E-01
rs4242084	rs2148575	CW, 1	0.50 (0.33 – 0.76)	1.31E-03	0.40 (0.21 – 0.75)	4.27E-03	0.47 (0.19 – 1.20)	1.15E-01	0.70 (0.34 – 1.41)	3.16E-01
rs10189905			0.62 (0.45 – 0.84)	2.46E-03	0.50 (0.34 – 0.74)	6.25E-04	0.56 (0.36 – 0.88)	1.21E-02	0.83 (0.56 – 1.21)	3.28E-01
rs17076974			1.29 (1.09 – 1.53)	2.64E-03	1.48 (1.19 – 1.84)	4.37E-04	1.32 (1.02 – 1.71)	3.48E-02	1.12 (0.90 – 1.39)	3.23E-01
rs7705993			1.30 (1.09 – 1.56)	4.13E-03	1.52 (1.22 – 1.88)	1.66E-04	1.30 (1.01 – 1.68)	4.04E-02	1.12 (0.90 – 1.39)	3.13E-01
rs3759607†			1.57 (1.14 – 2.15)	5.05E-03	2.03 (1.31 – 3.14)	1.46E-03	1.54 (0.89 – 2.67)	1.19E-01	1.21 (0.77 – 1.90)	4.15E-01
rs9436636			1.21 (1.06 – 1.39)	5.45E-03	1.34 (1.07 – 1.67)	1.10E-02	1.26 (0.97 – 1.63)	8.28E-02	1.07 (0.85 – 1.34)	5.61E-01
rs1544214			1.27 (1.07 – 1.50)	6.01E-03	1.41 (1.13 – 1.76)	2.30E-03	1.36 (1.05 – 1.76)	2.05E-02	1.08 (0.87 – 1.35)	4.83E-01
rs2129571			0.66 (0.49 – 0.89)	7.24E-03	0.63 (0.43 – 0.92)	1.65E-02	0.50 (0.30 – 0.81)	5.10E-03	0.87 (0.58 – 1.30)	4.90E-01
rs9857†			0.80 (0.68 – 0.94)	7.89E-03	0.70 (0.54 – 0.90)	6.34E-03	0.81 (0.58 – 1.14)	2.21E-01	0.91 (0.71 – 1.17)	4.59E-01
rs12155847*			1.31 (1.06 – 1.63)	1.38E-02	1.57 (1.24 – 2.00)	1.71E-04	1.30 (0.95 – 1.79)	1.06E-01	1.11 (0.89 – 1.40)	3.50E-01
rs13137776			0.81 (0.67 – 0.97)	2.32E-02	0.73 (0.58 – 0.93)	9.51E-03	0.74 (0.55 – 0.98)	3.67E-02	0.96 (0.76 – 1.22)	7.58E-01
rs10740308	rs2030057	CW, 0.95	0.73 (0.55 – 0.97)	2.83E-02	0.56 (0.42 – 0.75)	7.97E-05	0.80 (0.57 – 1.12)	2.02E-01	0.88 (0.65 – 1.18)	3.87E-01
rs7901888	rs2030057	CW, 1	0.73 (0.55 – 0.97)	2.83E-02	0.56 (0.42 – 0.75)	7.97E-05	0.80 (0.57 – 1.12)	2.02E-01	0.88 (0.65 – 1.18)	3.87E-01
rs7910196			1.25 (1.02 – 1.53)	3.37E-02	1.32 (0.98 – 1.78)	6.93E-02	1.47 (1.05 – 2.05)	2.63E-02	1.03 (0.75 – 1.40)	8.75E-01
rs231358	rs231355	CW, 0.96	1.20 (1.01 – 1.41)	3.42E-02	1.32 (1.06 – 1.64)	1.27E-02	1.29 (1.00 – 1.66)	5.06E-02	1.02 (0.82 – 1.27)	8.58E-01
rs9446187			1.48 (1.03 – 2.13)	3.45E-02	1.70 (1.17 – 2.46)	5.24E-03	1.92 (1.14 – 3.22)	1.36E-02	1.04 (0.69 – 1.57)	8.48E-01
rs2157673*			1.32 (1.02 – 1.72)	3.73E-02	1.55 (1.21 – 1.98)	4.44E-04	1.47 (1.08 – 2.01)	1.53E-02	1.04 (0.82 – 1.31)	7.42E-01
rs3793048	rs3806005	CW, 1	1.49 (1.02 – 2.18)	3.97E-02	1.76 (1.17 – 2.65)	6.60E-03	1.89 (1.12 – 3.19)	1.65E-02	1.04 (0.69 – 1.57)	8.47E-01
rs10762236			0.73 (0.54 – 0.99)	4.15E-02	0.55 (0.42 – 0.73)	3.02E-05	0.84 (0.60 – 1.17)	2.94E-01	0.87 (0.65 – 1.17)	3.62E-01
rs238272	rs9525613	CW, 1	1.25 (1.01 – 1.56)	4.24E-02	1.35 (1.05 – 1.72)	1.82E-02	1.46 (1.12 – 1.92)	5.59E-03	1.02 (0.81 – 1.29)	8.65E-01
rs7742508			1.55 (1.02 – 2.36)	4.24E-02	1.95 (1.26 – 3.03)	2.83E-03	1.91 (1.09 – 3.35)	2.45E-02	1.05 (0.68 – 1.61)	8.41E-01
rs17716275			1.61 (1.01 – 2.56)	4.53E-02	1.81 (1.07 – 3.07)	2.61E-02	2.19 (1.19 – 4.05)	1.22E-02	0.93 (0.45 – 1.90)	8.32E-01
rs7671659	rs6857038	P, 0.95	1.29 (1.00 – 1.67)	4.89E-02	1.54 (1.17 – 2.02)	1.89E-03	1.38 (0.98 – 1.94)	6.75E-02	1.01 (0.73 – 1.38)	9.76E-01
rs3998860			0.75 (0.56 – 1.00)	5.22E-02	0.57 (0.43 – 0.76)	8.52E-05	0.85 (0.61 – 1.19)	3.44E-01	0.89 (0.67 – 1.19)	4.27E-01
rs1615197*			1.33 (1.00 – 1.77)	5.28E-02	1.29 (1.04 – 1.60)	2.23E-02	1.82 (1.35 – 2.46)	9.69E-05	1.05 (0.84 – 1.31)	6.72E-01
rs7913568	rs3998860	CW, 0.97	0.75 (0.56 – 1.01)	5.75E-02	0.57 (0.43 – 0.76)	8.52E-05	0.85 (0.61 – 1.19)	3.44E-01	0.90 (0.67 – 1.21)	4.85E-01
rs7751843	rs7765440	CW, 1	1.45 (0.98 – 2.14)	6.39E-02	1.80 (1.25 – 2.59)	1.66E-03	1.74 (1.13 – 2.66)	1.11E-02	0.98 (0.67 – 1.44)	9.16E-01
rs1917527†	rs12635482	CW, 0.93	0.86 (0.73 – 1.01)	6.65E-02	0.75 (0.60 – 0.94)	1.08E-02	0.86 (0.65 – 1.14)	2.85E-01	0.98 (0.78 – 1.23)	8.69E-01
rs10831415	rs10831416	CW, 1	1.21 (0.98 – 1.50)	8.08E-02	1.27 (1.02 – 1.57)	3.30E-02	1.45 (1.12 – 1.87)	4.84E-03	0.99 (0.80 – 1.23)	9.29E-01
rs7521242			0.86 (0.71 – 1.03)	9.40E-02	0.72 (0.58 – 0.90)	4.10E-03	0.89 (0.69 – 1.15)	3.91E-01	0.97 (0.78 – 1.21)	8.15E-01
rs1016030	rs10831417	CW, 0.96	1.22 (0.97 – 1.53)	9.66E-02	1.25 (1.01 – 1.56)	4.37E-02	1.49 (1.15 – 1.93)	2.31E-03	0.99 (0.79 – 1.23)	8.93E-01
rs7625204			1.28 (0.95 – 1.71)	1.00E-01	1.57 (1.20 – 2.05)	9.41E-04	1.38 (1.02 – 1.87)	3.67E-02	0.97 (0.75 – 1.26)	8.29E-01
rs9807521†	rs977160	CW, 1	1.20 (0.96 – 1.50)	1.09E-01	1.38 (1.07 – 1.78)	1.32E-02	1.31 (0.95 – 1.81)	9.59E-02	0.98 (0.76 – 1.26)	8.48E-01
rs2281122†			1.36 (0.92 – 2.00)	1.25E-01	1.32 (0.99 – 1.76)	5.56E-02	2.03 (1.40 – 2.96)	2.14E-04	0.98 (0.75 – 1.28)	8.86E-01
rs1524962†			0.81 (0.61 – 1.07)	1.30E-01	0.65 (0.50 – 0.84)	1.07E-03	0.78 (0.56 – 1.08)	1.28E-01	1.02 (0.80 – 1.29)	8.85E-01
rs1450092			0.80 (0.60 – 1.07)	1.35E-01	0.62 (0.46 – 0.84)	2.05E-03	0.79 (0.57 – 1.11)	1.74E-01	1.02 (0.77 – 1.35)	8.84E-01
rs2335451†	rs10875941	CWP,1	0.77 (0.54 – 1.10)	1.50E-01	0.78 (0.62 – 0.99)	4.51E-02	0.52 (0.36 – 0.75)	3.99E-04	1.05 (0.82 – 1.34)	7.13E-01
rs10875941†			0.76 (0.52 – 1.11)	1.50E-01	0.78 (0.62 – 0.99)	4.51E-02	0.50 (0.34 – 0.71)	1.66E-04	1.05 (0.82 – 1.34)	7.13E-01
rs2593270	rs4765531	CW, 1	0.79 (0.58 – 1.09)	1.57E-01	0.71 (0.55 – 0.92)	9.77E-03	0.64 (0.47 – 0.86)	2.93E-03	1.07 (0.85 – 1.36)	5.56E-01
rs2656824	rs4765531	CW, 1	0.79 (0.58 – 1.09)	1.57E-01	0.71 (0.55 – 0.92)	9.77E-03	0.64 (0.47 – 0.86)	2.93E-03	1.07 (0.85 – 1.36)	5.56E-01
rs2941528*			0.78 (0.56 – 1.10)	1.59E-01	0.75 (0.58 – 0.97)	2.68E-02	0.58 (0.41 – 0.81)	1.38E-03	1.07 (0.83 – 1.38)	6.04E-01
rs2196180	rs1560901	CW, 1	1.16 (0.93 – 1.45)	1.78E-01	1.37 (1.07 – 1.76)	1.33E-02	1.23 (0.92 – 1.63)	1.58E-01	0.95 (0.75 – 1.21)	6.87E-01
rs3861810	rs1560901	CW, 1	1.16 (0.93 – 1.46)	1.83E-01	1.37 (1.07 – 1.76)	1.33E-02	1.23 (0.92 – 1.63)	1.58E-01	0.95 (0.75 – 1.21)	6.73E-01
rs2273816*			1.32 (0.87 – 2.01)	1.87E-01	1.59 (1.16 – 2.18)	4.20E-03	1.69 (1.16 – 2.47)	6.17E-03	0.88 (0.65 – 1.20)	4.30E-01
rs1594887	rs1529806	CW, 1	1.16 (0.92 – 1.46)	1.97E-01	1.39 (1.09 – 1.79)	9.01E-03	1.20 (0.91 – 1.59)	2.03E-01	0.95 (0.75 – 1.21)	6.70E-01
rs2060411			1.15 (0.92 – 1.44)	2.27E-01	1.33 (1.04 – 1.70)	2.48E-02	1.25 (0.94 – 1.66)	1.22E-01	0.93 (0.73 – 1.18)	5.45E-01
rs7629574†			0.84 (0.62 – 1.13)	2.41E-01	0.66 (0.51 – 0.84)	1.02E-03	0.84 (0.61 – 1.15)	2.73E-01	1.06 (0.83 – 1.35)	6.22E-01
rs10429616			1.20 (0.88 – 1.63)	2.41E-01	1.50 (1.21 – 1.87)	2.56E-04	1.27 (0.97 – 1.65)	7.95E-02	0.91 (0.73 – 1.13)	4.07E-01
rs10778050†	rs4764738	CW, 0.93	1.20 (0.88 – 1.63)	2.45E-01	1.12 (0.90 – 1.39)	3.09E-01	1.70 (1.26 – 2.28)	4.58E-04	0.95 (0.76 – 1.18)	6.41E-01
rs17138705†			0.76 (0.47 – 1.22)	2.51E-01	0.54 (0.39 – 0.75)	2.70E-04	0.70 (0.44 – 1.11)	1.28E-01	1.13 (0.83 – 1.53)	4.51E-01
rs2492367			0.71 (0.39 – 1.29)	2.62E-01	0.42 (0.28 – 0.61)	5.61E-06	0.76 (0.51 – 1.13)	1.76E-01	1.13 (0.82 – 1.57)	4.56E-01
rs1349282			0.82 (0.58 – 1.16)	2.62E-01	0.82 (0.66 – 1.02)	8.15E-02	0.59 (0.46 – 0.77)	7.47E-05	1.12 (0.88 – 1.43)	3.45E-01
rs2418412			1.19 (0.87 – 1.64)	2.77E-01	1.52 (1.22 – 1.89)	1.91E-04	1.25 (0.96 – 1.62)	1.02E-01	0.90 (0.72 – 1.12)	3.43E-01
rs7863577			0.83 (0.59 – 1.18)	3.12E-01	0.64 (0.44 – 0.93)	1.82E-02	0.82 (0.50 – 1.34)	4.29E-01	1.13 (0.75 – 1.71)	5.47E-01
rs2657940			1.18 (0.85 – 1.64)	3.19E-01	1.46 (1.13 – 1.89)	4.21E-03	1.32 (0.97 – 1.80)	7.41E-02	0.86 (0.66 – 1.13)	2.70E-01
rs2265346	rs10973012	P, 1	0.84 (0.60 – 1.19)	3.24E-01	0.67 (0.46 – 0.96)	2.93E-02	0.77 (0.48 – 1.22)	2.66E-01	1.15 (0.79 – 1.68)	4.57E-01
rs2263901	rs2657940	1	1.17 (0.85 – 1.63)	3.32E-01	1.45 (1.13 – 1.88)	4.17E-03	1.30 (0.96 – 1.77)	9.54E-02	0.86 (0.66 – 1.13)	2.70E-01
rs17347854			0.80 (0.51 – 1.27)	3.44E-01	0.69 (0.51 – 0.93)	1.44E-02	0.58 (0.41 – 0.84)	3.88E-03	1.26 (0.92 – 1.72)	1.44E-01
rs312929			0.88 (0.64 – 1.21)	4.44E-01	0.72 (0.58 – 0.91)	5.06E-03	0.79 (0.60 – 1.03)	8.21E-02	1.23 (0.94 – 1.61)	1.36E-01

* case-only data in CARE and WOSCOPS.

† case-only data in WOSCOPS.

We have also investigated the association of SNPs that have been previously reported to be associated with differential response to statin therapy. We investigated a variation in HMGCR gene by combining the analysis of rs17238540 in the case-only studies of CARE and WOSCOPS, and of a SNP in LD with rs17238540 (rs16872523, r^2^ = 1) in PROSPER/PHASE. Our analysis indicated that the major allele of this variant in HMGCR had greater risk reduction from pravastatin therapy (synergy index = 1.48; 95%CI 0.96 to 2.28; P = 0.076). This finding is consistent with the report by Chasman et al. (8) and Krauss et al. (5) who found that the major allele is associated with greater LDL-C reduction by statin therapy. We have also investigated the rs7412 SNP in APOE. We had no data for this SNP (or an LD SNP) in the PROSPER/PHASE study. However, a combined analysis of the case-only studies of CARE and WOSCOPS found that the major allele of this SNP (G, allele frequency = 0.95 among CARE Caucasian cases) is associated with greater risk reduction from pravastatin therapy (synergy index = 1.49; 955CI 1.01 to 2.22, P = 0.047). This result is inconsistent with a previous report by Thompson et al. (9) who found that the minor allele of this SNP was associated with greater LDL-C reduction from atorvastatin therapy.

## Discussion

We have conducted a genome-wide association study designed to identify genetic variants associated with differential CHD event reduction by pravastatin therapy and found that event reduction by therapy differed according to genotype for a SNP in the DNAJC5B gene (P = 4.8×10^-7^). Heterozygotes of rs13279522 (∼26% of the population) had 45% event reduction in a combined analysis of CARE, WOSCOPS and PROSPER/PHASE, while major homozygotes (∼71% of the population) had 13% event reduction by pravastatin.

DNAJC5B encodes a DnaJ/Hsp40 family member protein (DNAJ homolog subfamily C member 5B, also known as CSPbeta). DnaJ/Hsp40 family proteins are co-chaperones characterized by a highly conserved 70 residue J-domain, homologous to the *E.coli* DnaJ domain. The J-domain directs the interaction of DnaJ/Hsp40 proteins with specific Hsp70 family proteins. The interaction of DnaJ/Hsp40 proteins with Hsp70 proteins activates the Hsp70 ATPase activity and directs Hsp70 proteins to specific sub-cellular compartments [16]. CSPbeta is a palmitoylated membrane protein closely related to CSP, another DnaJ/Hsp40 family protein encoded by DNAJC5 [17], [18]. CSP has been implicated in regulation of vesicular secretion of neurotransmitters and of insulin secretion in cultured cells [17], [19]. The biological function of CSPbeta is not known; however, the high similarity [17], (65% identical amino acids, 78% conserved) between CSP and CSPbeta could suggest a similar biological function.

**Figure 4:**
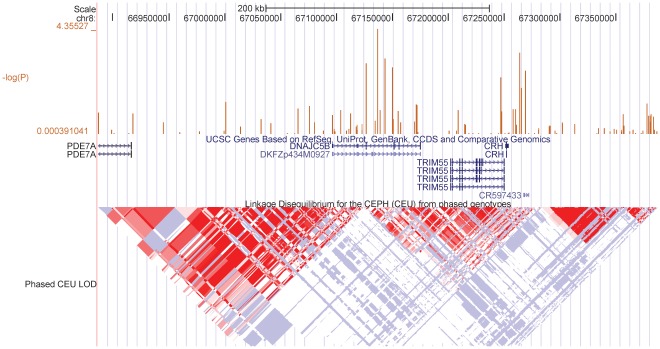
Genomic region of rs13279522. A ∼500 kb region centered around rs13279522 is from UCSC browser (http://genome.ucsc.edu). Linkage Disequilibrium (LD) information is color-coded at the bottom, genes (horizontal blue lines) and exons (vertical blue lines) are indicated above the LD representation. Vertical orange line correspond to the negative base 10 logarithm of the P value from a case only analysis of CARE and WOSCOPS for SNPs in this region. The location of rs13279522 is indicated by a black arrow.

Thus, DNAJC5B as well as other genes in the same linkage region (Figure 4), such as TRIM55 and CRH should be further investigated for potential functional role in modulation of event reduction by statin therapy.

We found no association between rs13279522 and baseline LDL-C levels or change in LDL-C levels by pravastatin therapy. If event reduction by pravastatin is at least partially mediated through non-LDL-C effects–such as reduction in protein isoprenylation and subsequent effect on inflammatory pathway [20], one could speculate that the rs13279522 variant is involved in these pleiotropic pathways. Alternatively, this variant could affect the susceptibility to high LDL-C levels, and thus be associated with individuals who benefit more from LDL-C reduction, as has been previously suggested for another genetic variant [21].

This study has several limitations. The case-only analysis used in the first stage of this investigation is valid only if genotype and treatment are independent of each other; however, in a randomized trial they are independent by design. The minimal P value observed in this is greater than the commonly used genome-wide significance threshold of 5×10^−8^. However, this observation is sufficiently intriguing to encourage other investigators to study this SNP in other randomized trials of statin therapy. This study was powered to detect SNPs associated with a 1.7 fold differential event reduction by pravastatin therapy, allele frequency greater than 0.15 at alpha = 10^−4^. Therefore, some SNPs were likely to be misclassified as non-associated SNPs (false negatives). Although one of the strengths of this study is that patients were randomized to 40 mg pravastatin therapy daily in all of the trials examined, the risk profile of the patients included in these studies varied. CARE is a secondary prevention trial which recruited patients who had had a myocardial infarction prior to the beginning of the study. WOSCOPS is a primary prevention trial who recruited patients with no history of myocardial infarction, but who had high risk profile for CHD. The PROSPER/PHASE study recruited patients who were elderly, either with or without history of CHD. Other potential differences between the studies, such as concomitant medication use and environmental exposures have not been accounted for. However, the randomized nature of these studies goes a long way toward addressing these potential biases.

In conclusion, in this genome wide association analysis of event reduction by pravastatin therapy, we have identified a SNP in the DNAJC5B gene that was associated with differential event reduction by pravastatin therapy in CARE, WOSCOPS, and PROSPER. Widespread replication of this association in multiple additional studies and potentially a clinical trial demonstrating improved outcomes or an investigation supporting the biological rationale for the mechanism of action of this variant are needed before the testing of this variant is introduced into clinical practice.

## Materials and Methods

### Study Design

This investigation was designed to identify genetic polymorphisms that were associated with differential CHD event reduction by pravastatin therapy. The individuals in the study were drawn from three randomized, placebo-controlled trials of pravastatin: CARE, WOSCOPS, and PROSPER/PHASE. We conducted the investigation in two stages (Figure 1). In the first stage, we identified genetic polymorphisms that were associated with differential event reduction by pravastatin therapy by conducting a GWAS among individuals who had CHD event during the CARE and WOSCOPS trials. In the second stage, polymorphisms that were nominally associated with differential event reduction (P<0.0001) were investigated in PROSPER/PHASE, and in an expanded analysis of CARE and WOSCOPS that added the included non-cases from these studies.

### Study Population

The population for this investigation was derived from three randomized, placebo-controlled trials of pravastatin: CARE, WOSCOPS, and PROSPER/PHASE. All patients in this investigation provided a written informed consent and the study protocol was approved by the institutional ethics review boards. CARE has been described previously [22]. Briefly, CARE was a secondary prevention double-blind trial of 4159 patients who had had a myocardial infarction between 3 and 20 months before randomization and had plasma total cholesterol levels below 240 mg/dL (3583 men and 576 women). These patients were randomly assigned to 40 mg of pravastatin per day or placebo. All laboratory measurements were made in a core laboratory. DNA in sufficient quantity and quality for this genetic investigation was available from 3109 CARE patients. In the first stage of this investigation, the CARE study population consisted of 682 patients who had a CHD event (a composite endpoint of fatal coronary event, nonfatal MI, or revascularization procedure) and whose whole genome scan data passed quality control. The second stage of this investigation included all CARE patients (those with and those without CHD events) who provided adequate DNA.

WOSCOPS has been previously described [23]. Briefly, WOSCOPS was a primary prevention double-blind trial of 6595 men, 45 to 64 years of age with no history of myocardial infarction and with mean plasma total cholesterol level of 272 mg/dL who were randomly assigned to 40 mg of pravastatin per day or placebo. All laboratory measurements were made in a core laboratory. DNA in sufficient quantity and quality for this genetic investigation was available from 5431 WOSCOPS patients. In the first stage of this investigation, the WOSCOPS study population consisted of 383 of these patients who had a CHD event (a composite endpoint of death from coronary heart disease, nonfatal MI, or revascularization procedures) and whose whole-genome scan data passed quality control. The second stage of this investigation included all WOSCOPS patients (those with and those without CHD events) who provided adequate DNA.

PROSPER has been previously described [24]. Briefly, PROSPER was a randomized, double-blind, placebo controlled trial of 5804 patients (2804 men, 3000 women) aged at least 70 years at enrollment with baseline total cholesterol levels that ranged from 155 mg/dL to 348 mg/dL. The trial included both patients with pre-existing vascular disease and those who were at risk for cardiovascular events due to smoking, hypertension, or diabetes. Patients were randomized to treatment with 40 mg pravastatin per day or placebo. All laboratory measurements were made in a core laboratory. DNA in sufficient quantity and quality for this genetic investigation was available from 5244 PROSPER patients who were genotyped in the genome-wide association analysis in the PROSPER/PHASE study. The primary endpoint was the composite endpoint of death from coronary heart disease, nonfatal MI, and occurrence of clinical stroke, either fatal or non-fatal.

### Genotyping

Genotyping for the whole-genome scan of the first stage was conducted using Infinium HumanOMNI-1 Quad V1 Beadchip from Illumina (San Diego CA) which interrogates 1.14 million markers. Quality control was performed according to Fellay et al. [22]. Briefly, we excluded 4 samples (1 from CARE and 3 from WOSCOPS) that had <99% call rate. The call rate was calculated after exclusion of: intensity-only markers (127,119), markers that did not achieve >99% call frequency (11,355), markers that had poor clustering (1,035) [25], Y chromosome markers that had >0% frequency in females (145), and X chromosome markers that had >1% heterozygote frequency in males (944). Genotyping reproducibility for 23 samples that were run in duplicates was >99.99%. In the second stage of this investigation, genotyping in CARE and WOSCOPS was performed by allele-specific PCR as previously described [26], the genotype concordance for samples that were genotyped by both methods was >99.8%. The genotyping data for the PROSPER/PHASE study was extracted from Illumina 660-Quad beadchips following manufacturer’s instructions. These beadchips contain 657,366 single nucleotide polymorphism (SNP) and copy number variants.

### Statistical Analysis

In order to make efficient use of the available resources for genotyping, in the first stage of this investigation, a case-only design was used to estimate whether event reduction during pravastatin therapy differed according to genotype. Since the genotype and treatment are independent of each other in a randomized clinical trial, the genotype frequencies among treated and untreated cases can be used to estimate the interaction between pravastatin treatment and genotype and to calculate the corresponding P value.

The P value for each SNP in the first stage of this investigation was evaluated in a logistic regression model with treatment status as the dependent variable and SNP coded in an additive model as the predictor variable. The rationale for this design is that if one of the alleles is associated with better event reduction by pravastatin treatment compared with placebo, we would expect that this allele frequency would be lower among cases of the treatment group compared with the placebo group. This expectation is justified if drug allocation is independent of genotype, a valid assumption by design in a randomized study. The odds ratio for the SNP from this logistic regression model was referred to as the synergy index by Davis et al. [15]. The regression model also included the following covariates: age for WOSCOPS and age, sex, and self reported ethnicity for CARE. The logistic regression models were fitted separately for CARE and WOSCOPS and the evidence for interaction from CARE and WOSCOPS was combined in a fixed effects model. The logistic regression and combined analyses of CARE and WOSCOPS were performed using PLINK version 1.07 [27].

In the second stage of this investigation, a time to event analysis was performed using Cox proportional hazards models and the Wald test was used to assess the effect of pravastatin, compared with placebo, on coronary events. Interaction between SNP and pravastatin treatment was assessed from models that included an interaction term between SNP and pravastatin therapy and included the following covariates: age for WOSCOPS, age, sex, and country of clinical center for PROSPER/PHASE, and age, sex, and principal components of the genetic variability using genotypes of 746 SNPs as previously described [28] for CARE. We used principal component analysis to exclude outliers (likely non-Caucasians) from the analysis of PROSPER/PHASE. Outliers were defined as those with a component value greater than 4 standard deviation away from the component value determined in the HapMap CEU population. R statistical software [29] was used for Cox regression models and for analyses that combined CARE, WOSCOPS, and PROSPER/PHASE studies.
